# Dataset of differentially expressed genes from SOX9 over-expressing NT2/D1 cells

**DOI:** 10.1016/j.dib.2016.08.047

**Published:** 2016-08-30

**Authors:** Louisa Ludbrook, Dimuthu Alankarage, Stefan Bagheri-Fam, Vincent Harley

**Affiliations:** a Centre for Reproductive Health, Hudson Institute of Medical Research, Melbourne, Australia; b Department of Biochemistry and Molecular Biology, Monash University, Melbourne, Australia

**Keywords:** SOX9, NT2/D1 cells, Microarray, Sex determination

## Abstract

The data presents the genes that are differentially up-regulated or down-regulated in response to SOX9 in a human Sertoli-like cell line, NT2/D1. The dataset includes genes that may be implicated in gonad development and are further explored in our associated article, “SOX9 Regulates Expression of the Male Fertility Gene Ets Variant Factor 5 (ETV5) during Mammalian Sex Development” (D. lankarage, R. Lavery, T. Svingen, S. Kelly, L.M. Ludbrook, S. Bagheri-Fam, et al., 2016) [1]. The necessity of SOX9 for male sex development is evident in instances where SOX9 is lost, as in 46, XY DSD where patients are sex reversed or in mouse knock-out models, where mice lacking Sox9 are sex reversed. Despite the crucial nature of this transcriptional activator, downstream target genes of SOX9 remain largely undiscovered. Here, we have utilized NT2/D1 cells to transiently over-express SOX9 and performed microarray analysis of the RNA. Microarray data are available in the ArrayExpress database (www.ebi.ac.uk/arrayexpress) under accession number E-MTAB-3378.

**Specifications Table**

**Table:** 

Subject area	*Biology*
More specific subject area	*Transcriptional regulation of human sex development*
Type of data	*Tables and figures*
How data was acquired	*Microarray analysis using Illumina HumanRef-8 v3.0 BeadChip*
Data format	*Processed, analyzed*
Experimental factors	*NT2/D1 cells were transiently transfected with SOX9 or empty vector*
Experimental features	*48 hours post transfection, total RNA was extracted according to the manufacturer׳s instructions (Qiagen) and hybridized to Illumina HumanRef-8 v3.0 BeadChip.*
Data source location	*Hudson Institute of Medical Research, Melbourne, Australia*
Data accessibility	*Data are within this article and microarray data are available in the ArrayExpress database (*www.ebi.ac.uk/arrayexpress*) under accession number E-MTAB-3378*

**Value of the data**

• This dataset provides a list of genes transcriptionally regulated by SOX9 during sex development in humans.
• SOX9 target genes are highly likely to play important roles within the testis as well as be mutated in disorders of sex development.
• Comparison of this dataset with other gonadal datasets can provide valuable insights into gonadal development.

## 1 Data

**Fig. 1:**
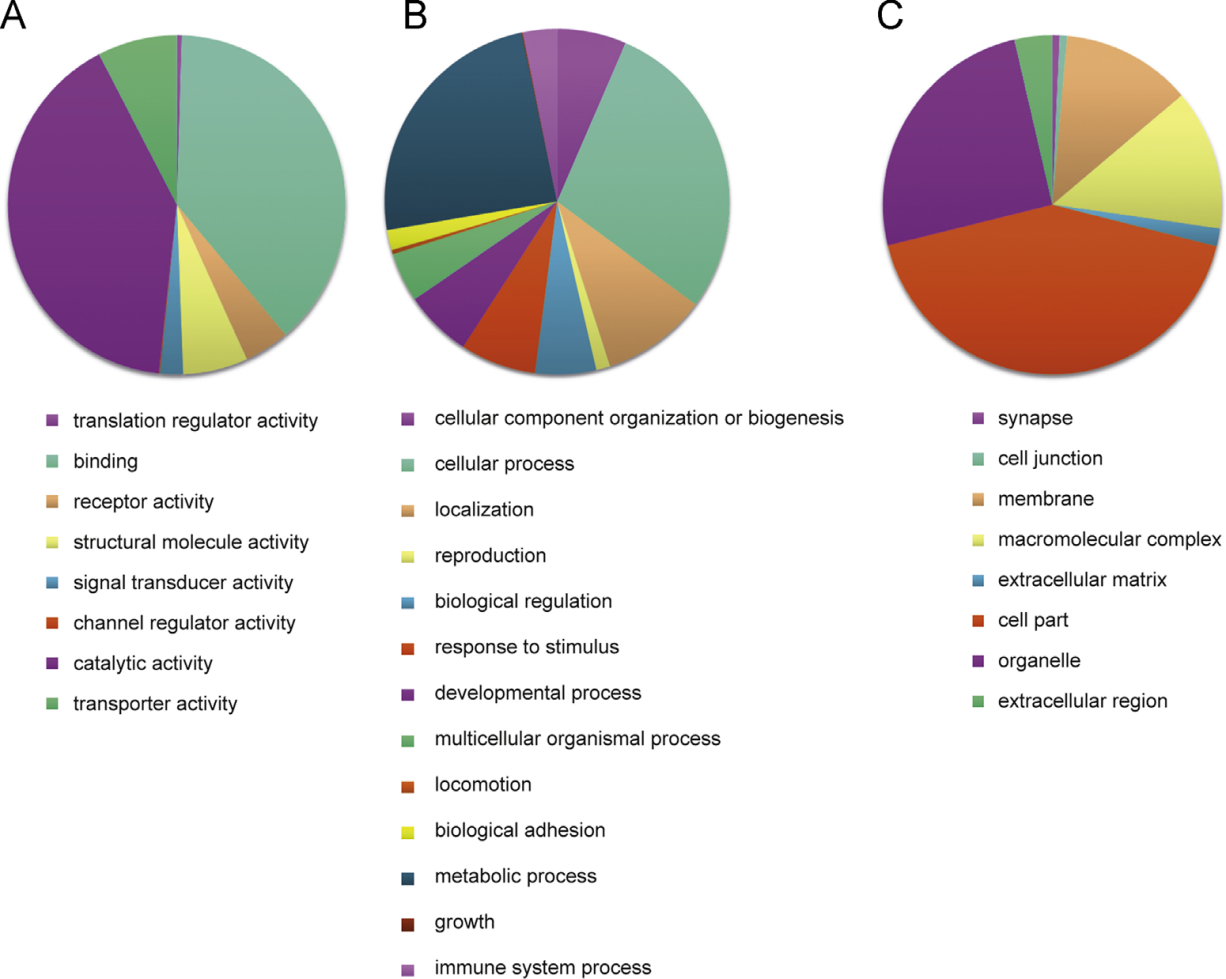
Distribution of GO terms in up-regulated genes associated with Molecular Function (A), Biological Processes (B) and Cellular Component (C).

**Fig. 2:**
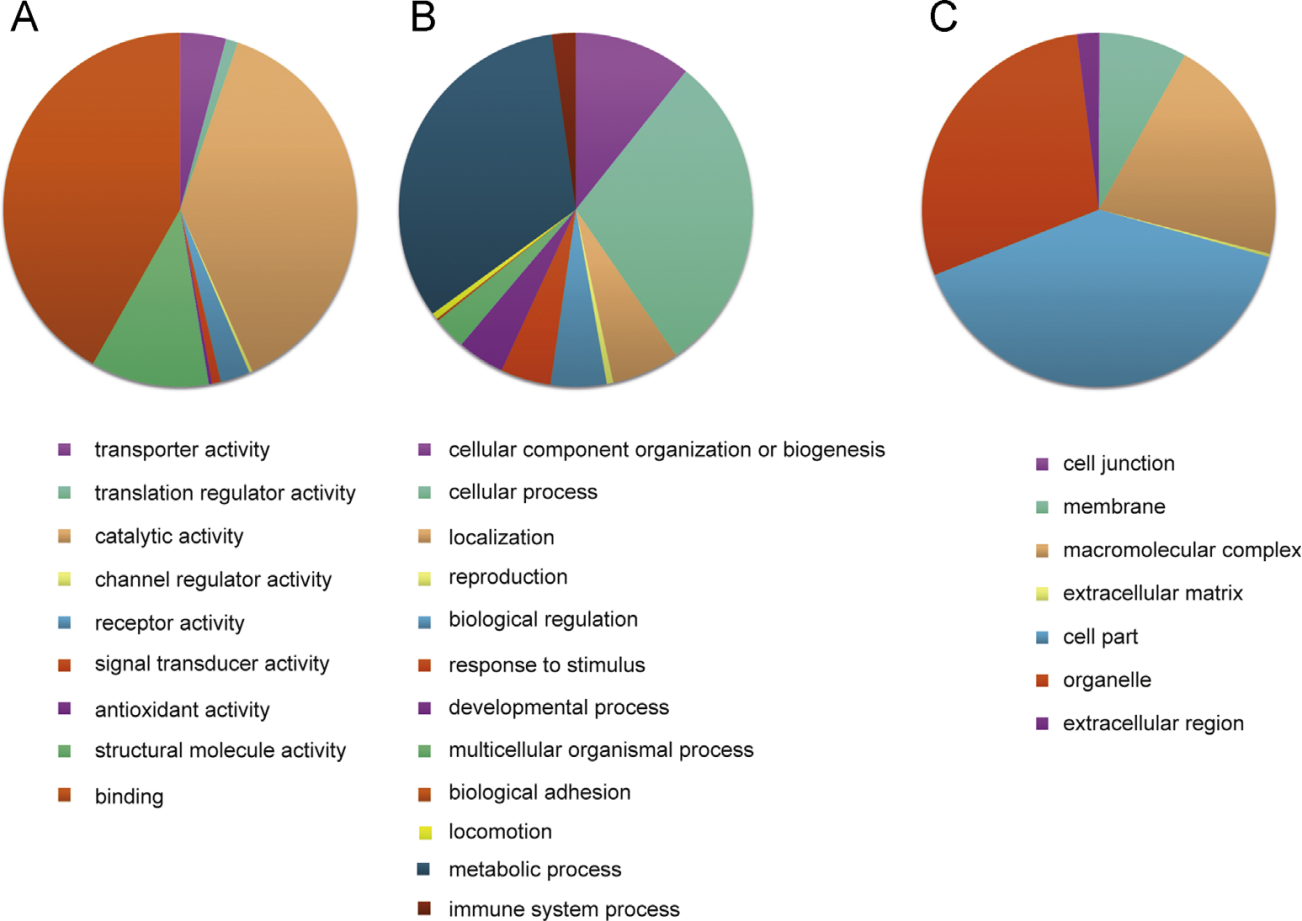
Distribution of GO terms in down-regulated genes associated with Molecular Function (A), Biological Processes (B) and Cellular Component (C).

Microarray analysis of gene expression in SOX9-NT2/D1 compared to vector-NT2/D1 identified 2626 differentially expressed transcripts with a±1.25 fold expression difference that was significant (*p*<0.05) (Supplementary Table 1). The 2626 genes account for ~10.7% of the total number of transcripts present on the Illumina BeadChip. Of the 2626 DEGs, 1312 transcripts were up-regulated (within a range of 1.25–2.35 fold change) and 1314 genes were down-regulated (within a range of −1.25 to −2.50 fold change), in response to SOX9 over-expression in the NT2/D1 cells. GO term analysis identified the most affected biological processes in up-regulated genes (Table 1) and in down-regulated genes (Table 2). The up and down regulated differentially expressed genes were annotated by association with three GO term categories: Molecular Function (MF), Biological Process (BP) and Cellular Component (CC) (Fig. 1, Fig. 2, Supplementary Table 2).

## 2 Experimental design, materials and methods

### 2.1 NT2/D1 transfection, RNA extraction and microarray analysis

Refer to the associated article [1] for detailed methods.

### 2.2 Data annotation

Gene ontology analysis of the differentially expressed genes was performed using PANTHER Overrepresentation Test and adjusted for multiple testing by Bonferroni correction (GO Ontology database Released 2016-06-22). A total of 231 GO terms were assigned to the up-regulated genes and 189 GO terms were assigned to the down-regulated genes (adjusted *p*-value <0.05). GO terms above *p*<0.05 were excluded from the analysis. Number of differentially expressed genes for particular GO terms was compared with total number of genes assigned to the term and enriched GO terms were highlighted (Table 1, Table 2). Differentially expressed genes that were up and down regulated were categorized to Molecular Functions, Biological Processes and Cellular Components (Fig. 1, Fig. 2, Supplementary Table 2).

## Figures and Tables

**Table 1:** The most affected GO terms from Biological Processes in up-regulated genes and their fold enrichment in SOX9 over-expressing NT2/D1 cells (adjusted *p*-value≤0.05).

Up-regulated genes		Total #	Fold enrichment
GO number	GO terms		
GO:0036498	IRE1-mediated unfolded protein response	15	4.62
GO:0030705	Cytoskeleton-dependent intracellular transport	21	4.07
GO:0000236	Mitotic prometaphase	23	4.01
GO:0002433	Immune response-regulating cell surface receptor signaling pathway involved in phagocytosis	19	3.86
GO:0038096	Fc-gamma receptor signaling pathway involved in phagocytosis	19	3.86
GO:0002431	Fc receptor mediated stimulatory signaling pathway	19	3.77
GO:0038094	Fc-gamma receptor signaling pathway	19	3.68
GO:0010970	Establishment of localization by movement along microtubule	20	3.52
GO:0016241	Regulation of macroautophagy	23	3.45
GO:0000819	Sister chromatid segregation	34	3.33
GO:0016925	Protein sumoylation	22	3.3

**Table 2:** The most affected GO terms from Biological Processes in down-regulated genes and their fold enrichment in SOX9 over-expressing NT2/D1 cells (adjusted *p*-value≤0.05).

Down-regulated genes		Total #	Fold enrichment
GO number	GO terms		
GO:0070125	Mitochondrial translational elongation	56	12.12
GO:0070126	Mitochondrial translational termination	55	11.62
GO:0001682	tRNA 5′-leader removal	7	11.56
GO:0016074	snoRNA metabolic process	7	11.56
GO:0006415	Translational termination	56	11.06
GO:0032543	Mitochondrial translation	59	10.12
GO:0006414	Translational elongation	62	9.01
GO:0042776	Mitochondrial ATP synthesis coupled proton transport	9	8.61
GO:0043624	Cellular protein complex disassembly	57	8.49
GO:0000291	Nuclear-transcribed mRNA catabolic process, exonucleolytic	12	7.03
GO:0043928	Exonucleolytic nuclear-transcribed mRNA catabolic process involved in deadenylation-dependent decay	11	6.89

## Appendix A Supplementary material

Supplementary material: Supplementary Table 1. Differentially expressed gene list derived from microarray of SOX9 over-expressing NT2/D1 cells compared to vector expressing NT2/D1 cells. Fold change cut-off is set at ±1.25 (*p*<0.05).

Supplementary material: Supplementary Table 2. GO analysis of the differentially expressed genes. Data are categorized by Biological Process (BP), Molecular Function (MF) and Cellular Compartment (CC).
